# Orbital-free photophysical descriptors to predict directional excitations in metal-based photosensitizers†
†Electronic supplementary information (ESI) available: Further computational details and additional tables. See DOI: 10.1039/d0sc01684e


**DOI:** 10.1039/d0sc01684e

**Published:** 2020-05-15

**Authors:** Pedro A. Sánchez-Murcia, Juan J. Nogueira, Felix Plasser, Leticia González

**Affiliations:** a Institute of Theoretical Chemistry , Faculty of Chemistry , University of Vienna , Währinger Str. 17 , 1090 Vienna , Austria . Email: pedro.murcia@univie.ac.at ; Email: leticia.gonzalez@univie.ac.at; b Department of Chemistry and Institute for Advanced Research in Chemistry , Universidad Autónoma de Madrid , Madrid , 28049 , Spain; c Department of Chemistry , Loughborough University , Loughborough , LE11 3TU , UK; d Vienna Research Platform for Accelerating Photoreaction Discovery , University of Vienna , Währinger Str. 17 , 1090 Vienna , Austria

## Abstract

The development of dye-sensitized solar cells, metalloenzyme photocatalysis or biological labeling heavily relies on the design of metal-based photosensitizes with directional excitations. Directionality is most often predicted by characterizing the excitations manually *via* canonical frontier orbitals. Although widespread, this traditional approach is, at the very least, cumbersome and subject to personal bias, as well as limited in many cases. Here, we demonstrate how two orbital-free photophysical descriptors allow an easy and straightforward quantification of the degree of directionality in electron excitations using chemical fragments. As proof of concept we scrutinize the effect of 22 chemical modifications on the archetype [Ru(bpy)_3_]^2+^ with a new descriptor coined “substituent-induced exciton localization” (SIEL), together with the concept of “excited-electron delocalization length” (EEDL_*n*_). Applied to quantum ensembles of initially excited singlet and the relaxed triplet metal-to-ligand charge-transfer states, the SIEL descriptor allows quantifying how much and whereto the exciton is promoted, as well as anticipating the effect of single modifications, *e.g.* on C-4 atoms of bpy units of [Ru(bpy)_3_]^2+^. The general applicability of SIEL and EEDL_*n*_ is further established by rationalizing experimental trends through quantification of the directionality of the photoexcitation. We thus demonstrate that SIEL and EEDL descriptors can be synergistically employed to design improved photosensitizers with highly directional and localized electron-transfer transitions.

## Abstract 2

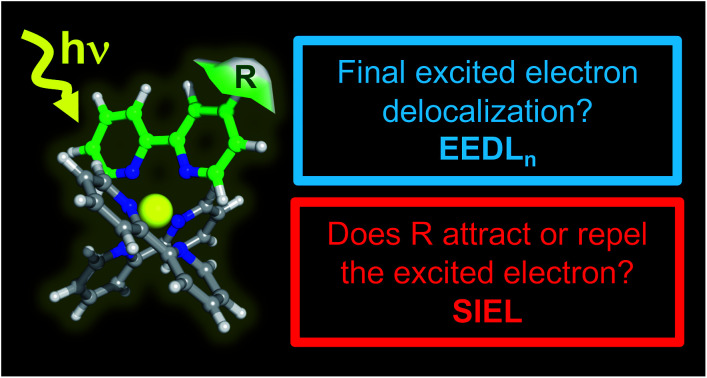
We report the descriptor *substituent-induced exciton localization,* which together with the *excited-electron delocalization length* concept, is able to quantify how functional groups affect the directionality of light-driven electronic excitations.

## Introduction

Transformation of light into chemical energy is one of the central challenges of this century. Inspired by nature, scientists are constantly searching for rules to design molecular devices made of chemical structures able to absorb light in a particular wavelength range. After light absorption, the excited electron may be transferred to an acceptor located in the surroundings from where it can reduce a third species,^1^,^2^ it can be stored as energy as in Grätzel cells,^3^,^4^ or it might evolve through other paths.^5^ These electron-transfer and charge-separation processes can be highly directional and are controlled by the chemical nature of the donor and acceptor species,^6^–^8^ their relative spatial orientation within the optical device, and the environmental conditions.

**Fig. 1:**
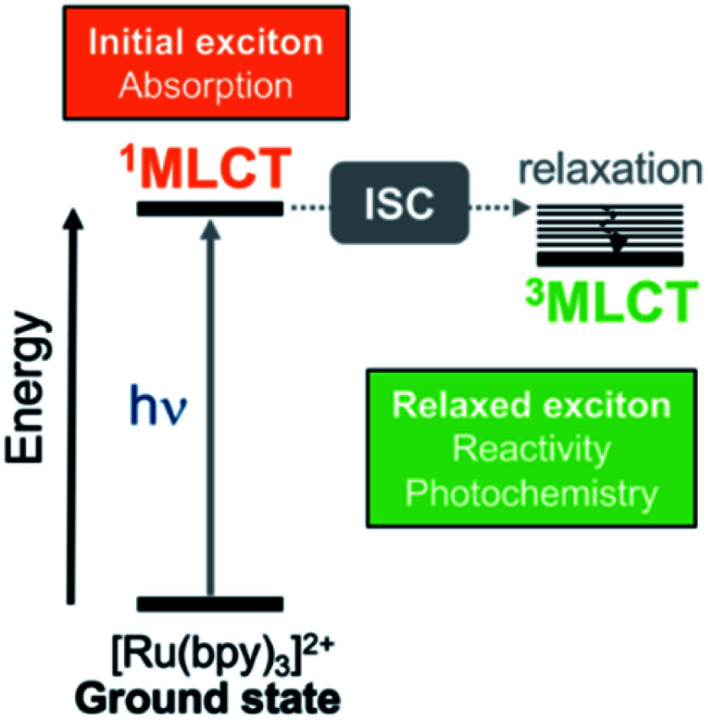
Simplified Jablonski diagram that shows the initially excited singlet metal-to-ligand charge-transfer (^1^MLCT) and relaxed triplet metal-to-ligand charge transfer (^3^MLCT) states in [Ru(bpy)_3_]^2+^. The relaxed ^3^MLCT state is populated after ultrafast intersystem crossing (ISC)^17^–^19^ from ^1^MLCT^20^ and then, subsequent vibrational relaxation can take place.

A prototypical chromophore employed in charge-separation experiments is [Ru(bpy)_3_]^2+^. It presents a long-lived (*ca.* 1 μs in solution)^9^ triplet metal-to-ligand charge transfer (^3^MLCT) state from which electron transfer can further evolve, as sketched in Fig. 1. The exciton picture (hole + excited electron) of [Ru(bpy)_3_]^2+^ is highly dynamical, *i.e.* it changes upon light absorption, so that *e.g.* whereas in the initial and short-lived ^1^MLCT state the exciton is delocalized, after evolution to the ^3^MLCT the excited electron is localized on only one unit.^10^ The effect of chemical modifications on the electron excitations within chromophores like [Ru(bpy)_3_]^2+^ has been traditionally analyzed by inspecting the canonical frontier orbitals HOMO and LUMO.^11^–^15^ General trends have been formulated in coordination complexes, such that electron-withdrawing groups (EWG) located on the ligands tend to have a stronger stabilizing effect on the ligand-centered orbitals (usually LUMO) than on the metal-centered orbitals (HOMO). In contrast, electron donating groups (EDG) behave opposite and destabilize more the HOMO with respect to the LUMO.^11^,^16^ Although widespread, this traditional approach is an oversimplification that neglects that, more often than not, electronic excitations involve more than one orbital, complicating the interpretation. This scenario is even more intricate when the number of calculations increases, *e.g.*, if an ensemble of structures is considered to account for nuclear vibrational energy or if a large sampling of geometries is required to describe the chromophore within an explicit environment, or if the analysis is to be done within a time-resolved simulation. In such cases, a characterization by visual inspection of orbitals is a very time-consuming process or simply unaffordable, let alone be quantitative.

In order to circumvent these limitations, universal orbital-free molecular descriptors are highly commendable and a large body of groups have worked on the development of density-based descriptors in the last decade.^21^–^33^ Particular effort has been spent on quantifying the overall amount of charge transfer including its effect on excitation energies.^34^,^35^ However, a more fine-grained picture is advantageous for multichromophoric systems, such as transition metal complexes, where a fragment-based analysis approach^36^,^37^ was shown to be particularly powerful.^38^ In this work, we extend the reach of this toolbox by introducing a new photophysical descriptor based on the analysis of the one-electron transition-density matrix:^23^,^38^,^39^ the substituent-induced exciton localization (SIEL), and showing its power in real-life situations. We show that, allied with the excited-electron delocalization length (EEDL_*n*_)^36^,^40^ definition, it is straightforward to quantify and predict the effect that chemical functionalization has on exciton populations using the familiar chemical concept of building blocks and thereby eliminating molecular orbitals.

While the EEDL_*n*_ measures over how many fragments or ligands (for instance a bipyridyl ligand) the excited electron is delocalized, SIEL predicts quantitatively how the presence of a functional group affects the electron population in a ligand of the coordination sphere. The power of this approach is showcased on the archetypical [Ru(bpy)_3_]^2+^ modified with 22 functional groups R as a systematic platform of study. The descriptors are used to quantify the effect that a particular functional group has on the localization of both initially excited singlet and relaxed triplet states within a quantum distribution of geometries that accounts for nuclear vibrational motion. Finally, we illustrate the general predictiveness of SIEL with in four different experimental playgrounds where the directional excitation process determines the properties of the photosensitizer.^41^–^46^

## Systems under study

**Scheme 1:**
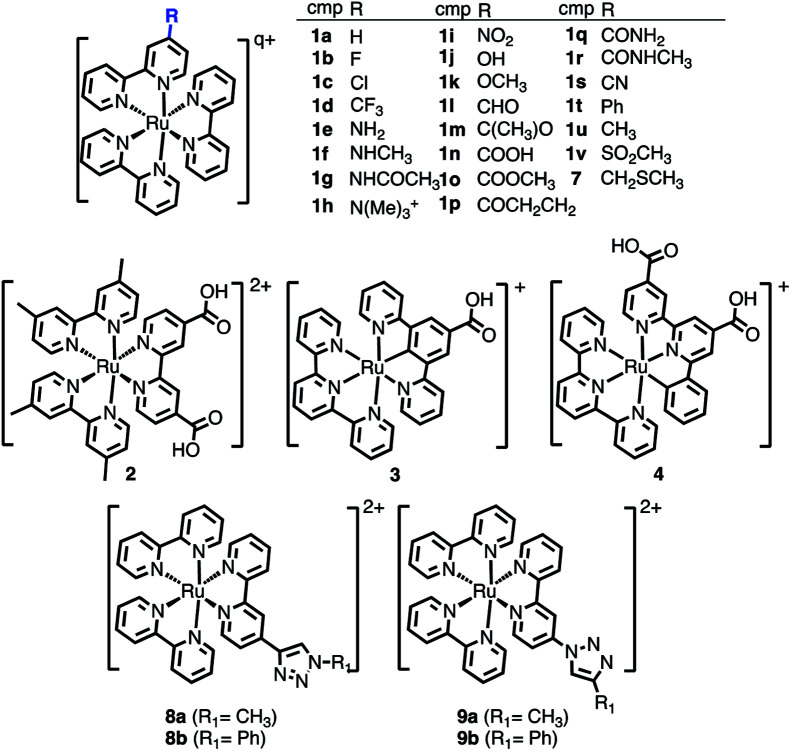
Chemical derivatives **1b–1v** of [Ru(bpy)_3_]^2+^ (**1a**), **2–4**, and **7–9** considered in this work. In the table, total charge *q* = 2 for all compounds except for **1h** (*q* = 3).

The selected chemical modifications on [Ru(bpy)_3_]^2+^ (**1a**) are shown in Scheme 1. Most of them correspond to synthetically accessible modifications with EWGs and EDGs in the 4-position of one bpy unit of [Ru(bpy)_3_]^2+^ (Table S1†) and many of them are chemically interconvertible by standard chemical transformations. We have explored a halogen series (**1b–1d**), an amine series where the amine group (**1e**) is methylated (**1f**), permethylated (**1h**), acetylated (**1g**) and oxidized to NO_2_ (**1i**), a hydroxyl/carbonyl series with a hydroxyl group (**1j**) and its methylated form (**1k**) as well as the oxidized aldehyde (**1l**), ketone (**1m**), carboxylic acid (**1n**), methyl ester (**1o**), amide (**1q**) and methyl amide (**1r**). In addition, in this series a nitrile **1s** (precursor after hydrolysis of an amide) and the α,β-unsaturated carbonyl **1p** are included. Finally, the phenyl ring (**1t**), the methyl group (**1u**) and the methyl sulfone (**1v**) are also evaluated.

Additionally, the [Ru(dmb)_2_(dcb)]^2+^ (**2**), the carbometallated Ru-complexes [Ru((COOH)-N∧C∧N)(tpy)]^+^ (**3**) and [Ru((COOH)_2_-C∧N∧N)(tpy)]^+^ (**4**),^42^ the Ru–diimine complex [Ru(bpy)_2_(bpy-CH_2_SCH_3_)]^2+^ (**7**)^43^ and two click-chemistry products,^45^,^46^ **8a–8b** and **9a–9b**, were considered for study, see Scheme 1.

## Theory

### Computational details

For each compound we have computed the exciton properties of the initially excited ^1^MLCT state as well as of the relaxed ^3^MLCT state obtained after intersystem crossing (ISC) (recall Fig. 1). Whereas the initial exciton is responsible for the absorption properties of the complex, the latter exciton is key for emission and the subsequent photochemistry of the triplet CT state. Each complex was optimized in the ground state and in the first triplet state by means of density functional theory (DFT) and its time-dependent version TD-DFT. Then, a quantum ensemble of 100 geometries at 300 K was considered for each of the spin cases (^1^MLCT and ^3^MLCT) to account for an appropriate conformational sampling due to nuclear vibrational energy.^38^,^40^,^47^,^48^ The computation of the ^1^MLCT absorption band involved the lowest 25 singlet states for each geometry (*i.e.* 25 × 100 geometries = 2500 excited states per derivative) and the computation of the ^3^MLCT emission band involved 1 state for each geometry (*i.e.* 1 × 100 geometries = 100 excited states per derivative). Since the ^3^MLCT is a manifold of states close in energy, a total of three excited triplet states per complex was first explored for statistical significance (see the Boltzmann weighting, Table S4†) before computing the emission spectrum.

All the electronically excited-state energies and properties in the different ensembles were computed by TD-DFT within the Tamm–Dancoff approximation (see Section S1† for further computational details).

### Definition of descriptors

**Fig. 2:**
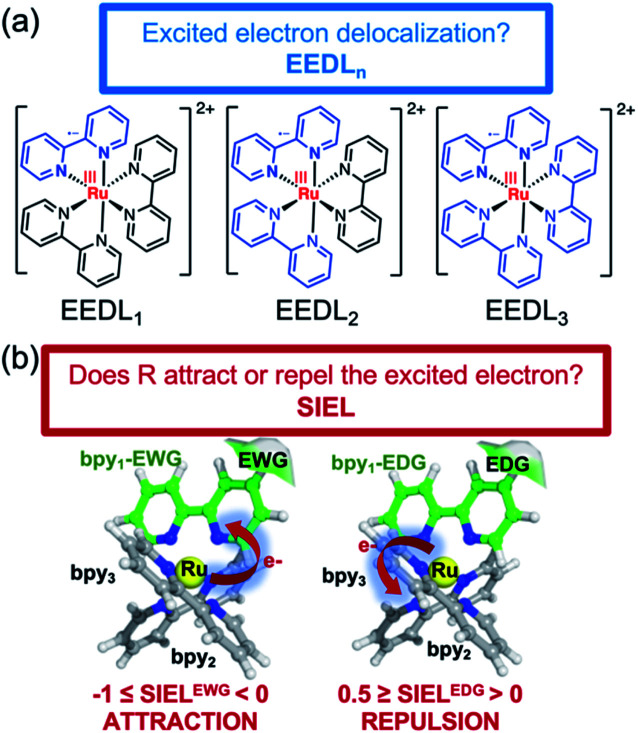
Illustration of EEDL_*n*_ (a) and SIEL (b) descriptors using [Ru(bpy)_3_]^2+^. In (b) an electron-withdrawing group (EWG) or electron donating group (EDG) functional group in the bpy1 ligand is sketched to indicate the electron directionality (arrow).

The excited electron delocalization length on *n*-fragments (EEDL_*n*_), introduced elsewhere,^40^ is defined as the percentage of the total excited electron population that is localized on *n*, where *n* = 1, 2, 3 or 4 fragments, regardless of which one it is. It is computed based on the final partition ratio (PR_f_) defined as:1
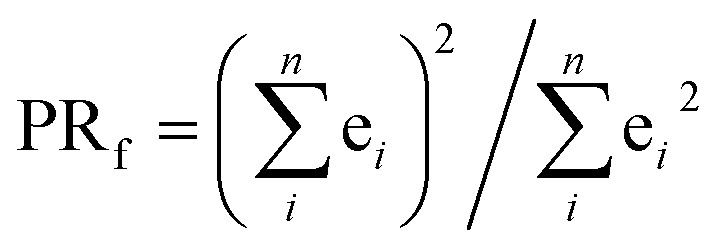
where e_*i*_ is the excited electron population on the fragment *i*.^23^,^38^,^39^ For example, the EEDL_1_ value (*n* = 1), will be computed in percentage as the total number of states with PR_f1_ over the sum of the total excited states (PR_f1_ + PR_f2_ + PR_f3_ + PR_f4_). In general, we define EEDL_*n*_ as,

2 EEDL_*n*_ = *N*(PR_f*n*_)/*N* × 100, %

where *N*(PR_f*n*_) is the number of excited states with PR_f*n*_ defined as *n* – 0.5 < PR_f_ < *n* + 0.5 and *N* the total number of excited states.^23^ EEDL_*n*_ is calculated by means of an electronic population analysis that quantifies how the excited electron is distributed over the different fragments *n*.^36^ In other words, EEDL_*n*_ allows discriminating between the case where all excited states are localized on one ligand (*n* = 1) and thus EEDL_1_ is close to 100% and a delocalized excited electron where EEDL_2_, EEDL_3_ or EEDL_4_ would present values larger than 0, indicating certain degree of delocalization over 2, 3 or 4 fragments, respectively (see Fig. 2a). In principle, for MLCT states, the population of the excited electron on the metal center is not significant and EEDL_4_ is close to zero.

The new descriptor SIEL reports how a particular substituent R attracts or repels the excited electron into the ligand where this functional group R is located. SIEL is computed as a weighted sum of the population of the excited electron (e^*R*^) on the ligand where the substituent is located, bpy_1_-R, and the population of the excited electron over all other ligand fragments (e_*i*_):3
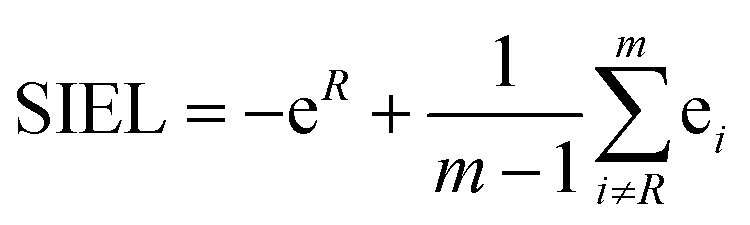
where *m* is the number of fragments excluding the metal center (*i.e.* in this case *m* = *n* – 1). By definition, for three ligands the SIEL descriptor takes values between 0.5 and –1 depending on the effect of the chemical substituent (in the general case with a different number of ligands, the upper boundary would be 1/(*m*^–1^)). It quantifies how an EWG attracts the excited electron (–1 ≤ SIEL < 0) or how an EDG repels it (0.5 ≥ SIEL > 0), see Fig. 2b. The factor in front of the sum is chosen such that an evenly delocalized state yields SIEL = 0, allowing for quantification in any metal complex with more than 3 ligands by customization of the value of *m*. If the metal center is not included in the analysis of the final population of the excited electron, *m* will be equal to *n* (*n* value used for EEDL_*n*_).

The automatized electronic-structure analysis to compute EEDL and SIEL was implemented within the program package TheoDORE.^37^ The computational protocol to compute EEDL_*n*_ and SIEL numbers is described in Section S2 of the ESI.† For each of the [Ru(bpy)_3_]^2+^ compounds **1b–1v**, the system was divided into four fragments: the metal center and the three bpy ligands. The same fragment definition was applied to those compounds based on the same scaffold. For **3** and **4**, three fragments were defined.

## Results and discussion

### Excited-electron delocalization length (EEDL_*n*_) on [Ru(bpy)_3_]^2+^ derivatives

**Fig. 3:**
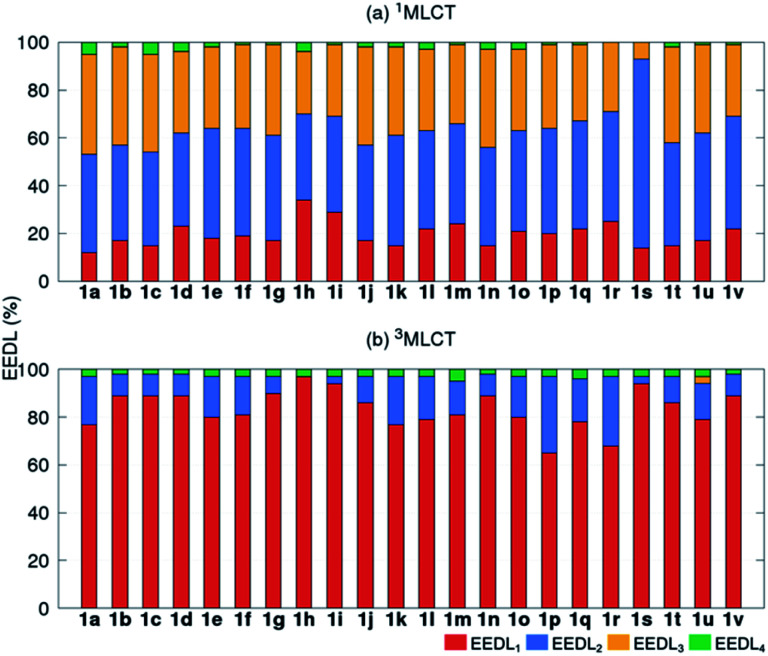
Mean EEDL_*n*_ values (%) for **1a–1v** [Ru(bpy)_3_]^2+^ derivatives in the (a) ^1^MLCT (averaged over 2500 states) and (b) relaxed lowest ^3^MLCT (averaged over 100 states) bands.

We start the discussion with the EEDL_*n*_ values of the 22 derivatives of [Ru(bpy)_3_]^2+^**1a–1v** (recall Scheme 1), collected in Fig. 3 as percentage bars (see also Tables S2 and S3†). All 2500 excited states of the prototype **1a** (R = H) indicate that the singlet excited electron is mainly delocalized over two (EEDL_2_ = 44%, blue bar, Fig. 3a) and three bipyridine units (EEDL_3_ = 43%, yellow bar). Only 15% of the states are localized on one ligand (EEDL_1_, red bar) and a very small fraction of states are delocalized over the three ligands and the metal center (EEDL_4_ = 4%, green bar). These numbers agree well with experimental results that confirm initial excited electron delocalization in the excited singlet state.^10^

The introduction of EWG or EDG affects to a small extent the net localization of the initial excited electron compared to **1a** (compounds **1b–1v**, Fig. 3a). In all cases, the excited electron is mainly delocalized over 2 bpy ligands (blue bars) with significant (but smaller) contributions from EEDL_1_ (red bar) and EEDL_3_ (yellow bar). Only the presence of strong EWG groups like N(CH_3_)_3_^+^ (**1h**) and NO_2_ (**1i**) increases the population of the singlet excited electron on only one bpy unit with values for EEDL_1_ of *ca.* 30%.

In the relaxed ^3^MLCT state of [Ru(bpy_3_)]^2+^, which is formed after ISC and vibrational relaxation, the exciton has been proven experimentally to be localized.^17^,^18^,^49^ This is confirmed in our analysis that predicts EEDL_1_ larger than 65% (red bar, Fig. 3b) and a delocalization over two ligands of less than 25%. This analysis clearly evidences how the electronic distribution strongly changes between the initial singlet and relaxed triplet excited states. It is worth to stress that such changes would barely be predictable and quantified within a simplified inspection of HOMO–LUMO orbitals.

### Substituent-induced exciton localization (SIEL) on [Ru(bpy)_3_]^2+^ derivatives

**Fig. 4:**
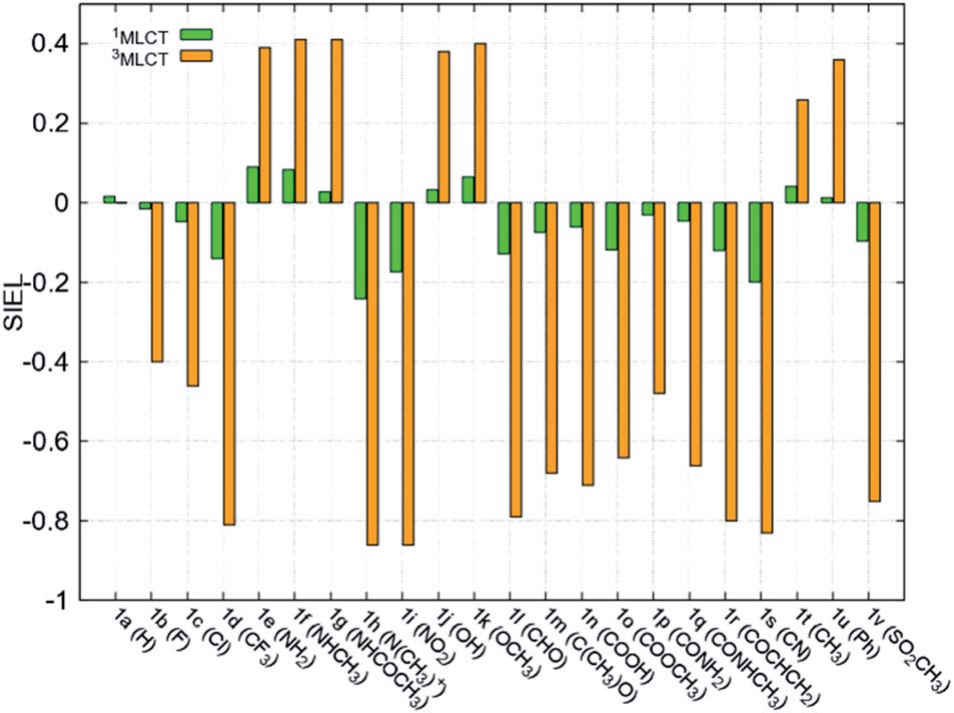
Mean SIEL values for the initial singlet excited band (^1^MLCT, green bars, averaged over 2500 states) and the relaxed triplet excited band (^3^MLCT, orange bars, averaged over 100 states).

Fundamental further insight is provided by the SIEL descriptor, which reveals where the excited electron is directed. The SIEL values of **1a–1v** are plotted in Fig. 4a for both the singlet and triplet states (see also Table S5†). The parent compound **1a** shows SIEL values in both singlet and triplet manifolds close to 0 because all three ligands are the same (R = H). In contrast, SIEL in **1b–1v** beautifully illustrates how the chemical nature of the substituent determines the directionality of the excitation, in both singlet and triplet states. It could be argued that the general trends could have been expected from chemical intuition: the EWGs (*e.g.* all the halogens (**1b–1d**), N(CH_3_)_3_^+^ (**1h**), NO_2_ (**1i**) or CN (**1s**)) attract (SIEL < 0) and EDGs (*e.g.* all the amines (**1e–1g**), OH (**1j**) or OCH_3_ (**1k**)) repel (SIEL > 0) the excited electron. However, the use of SIEL is much more powerful than simple chemical sense, as *e.g.* it allows to identify subtle and not so obvious differences within a chemical family. For instance, all the neutral amine derivatives (**1e–1g**) have positive SIEL values, but whereas the alkylation of the amine (**1e** → **1f**) almost does not affect the SIEL value in the ^1^MLCT state, the acetylation in **1g** reduces the SIEL value compared to **1e** (green bars, Fig. 4). Remarkably, full methylation in **1h** changes the sign of SIEL. Within the carbonyl series (**1l–1r**), the increase of the oxidation state on the carbonyl carbon reduces the SIEL absolute value. Whereas the aldehyde **1l** and the methyl ester **1o** show a large negative SIEL value, the amide **1p** show smaller negative SIEL values. Interestingly, in the ^3^MLCT state, the methylation of the amide (**1q**) increases the attraction of the excited electron in the same extend as a in the methyl ester **1o**. The NO_2_ (**1i**) and N(CH_3_)_3_^+^ (**1h**) derivatives show the largest negative SIEL values of all the compounds.

**Fig. 5:**
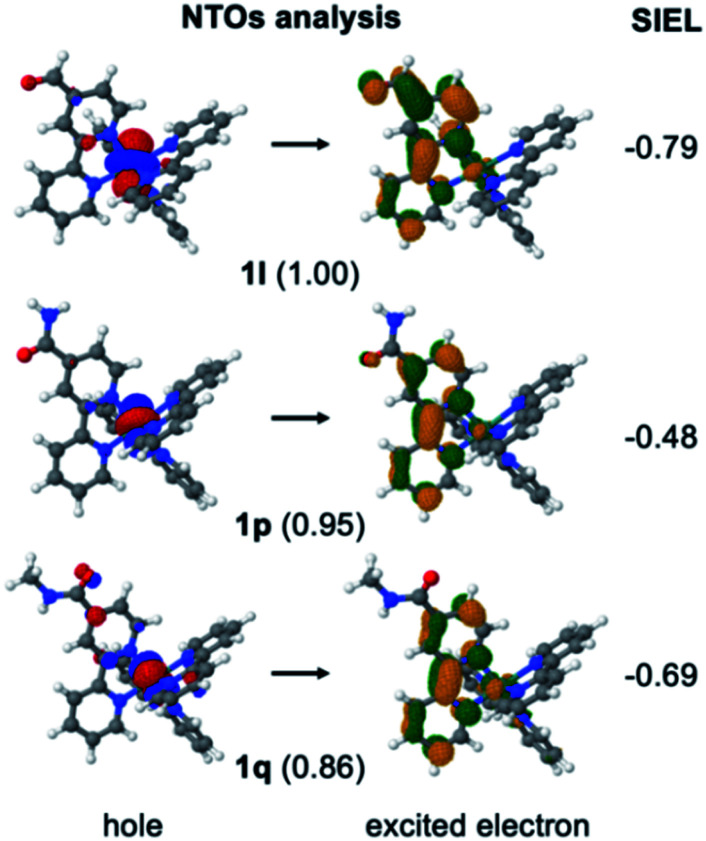
Natural transition orbitals (NTOs) computed in the minimum energy geometry of the ^3^MLCT state of **1l**, **1p** and **1q**. The weights are shown in parenthesis. Mean SIEL average values of bpy1-R (over 100 geometries) of the lowest ^3^MLCT state of **1l**, **1p** and **1q** are shown on the right.

In order to stress the virtues of the SIEL descriptor against traditional orbital inspection, we plot the corresponding natural transition orbitals (NTOs) of the former **1l**, **1p** and **1q** compounds in Fig. 5. The aim is to try to explain the effect of the substituent on the electronic excitation of the ^3^MLCT state. However, we can see that in all three cases the excited electron is localized on bpy1 – the ligand that bears the functional group. Also, in the three cases the hole comes from the metal center, and so all three compounds evince identical MLCT character. How then to differentiate amid the three cases? Which is the stronger electron acceptor involved in the excitation? Clearly, the weights for the NTOs do not help either, as all of them are similar, representing over 85% of the excitation. In contrast, the shrewd SIEL descriptors can be introduced and applied to revelatory effect (Fig. 5): the population of the excited electron in the ^3^MLCT state evinces a clear increase in bpy1-R in the order **1p** < **1q** < **1l**.

**Fig. 6:**
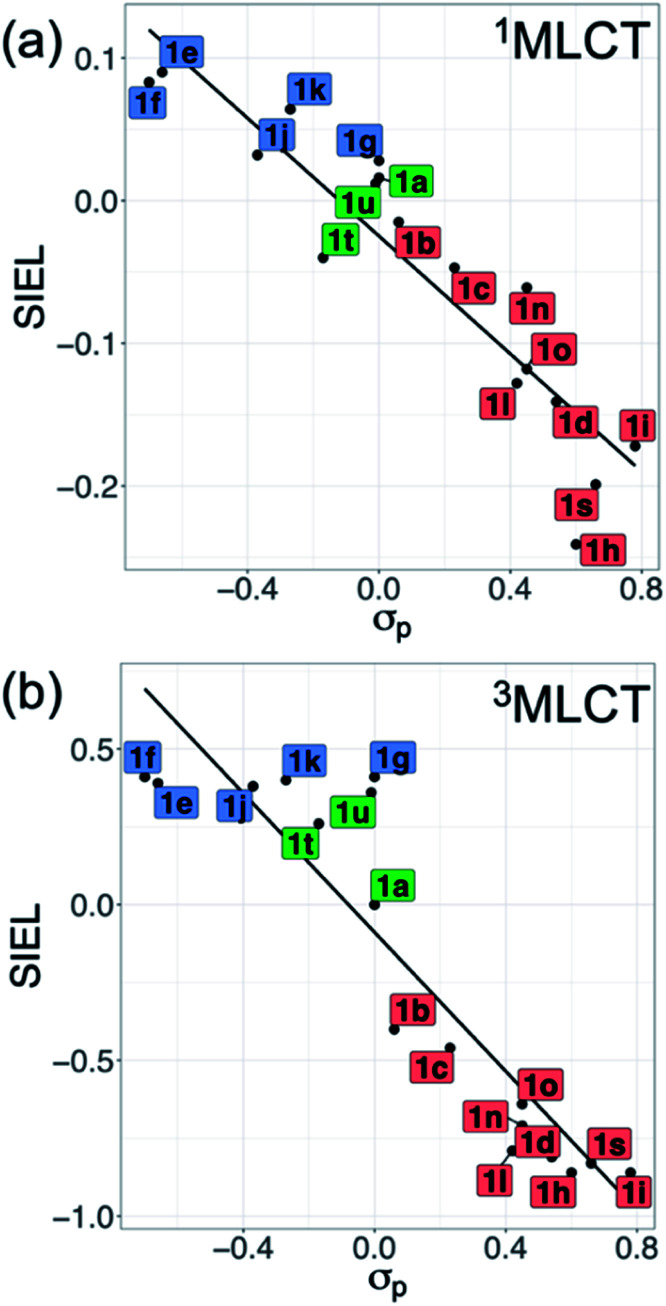
Correlation plots of averaged SIEL values of the (a) vertical ^1^MLCT and (b) relaxed ^3^MLCT states with *σ*_p_. Labels of **1a–1v** are colored by EWG (red), EDG (blue) and H/alkyl/aryl (green) groups. No tabulated data were found for **1m**, **1p**, **1q** and **1v**.

Also revealing are the differences between the SIEL values for the singlet and triplet states (Fig. 4). Although the sign of SIEL for each functional group is the same in both ^1^MLCT and ^3^MLCT states, the values are different: the absolute values are larger in the triplet state, as expected from the EEDL_*n*_ values. This confirms that caution should be exercised when considering computed properties at the Franck–Condon region to explain the behavior of the electronic states beyond this region (*e.g.* at the relaxed triplet state). As an example, we compare the SIEL descriptor with the empirical substitution constants of Hammet (*σ*_p_)^50^ and the electrophilic substituent constants of Brown and Okamoto (*σ*_p_^+^)^51^ (Table S5†) – descriptors that have been previously used to describe the substituent effect on the photophysics of polypyridine Ru-based complexes.^52^–^54^ The best correlation was found with *σ*_p_, plotted in Fig. 6a and b, for both SIEL values of the initial ^1^MLCT band (*R*^2^ = 0.841) and of the relaxed ^3^MLCT states (*R*^2^ = 0.829), respectively. However, since these *σ*_p_ parameters have been established for the electronic ground state, they cannot discriminate between the electronic behavior in the initial and the final relaxed exciton. SIEL does. For example, it shows larger absolute values for states localized on one ligand and smaller values for states largely delocalized. Even more, compared to the empirical parameters, SIEL can reflect for a particular functional group not only its electronic nature – as proven above – but also the effect of the environment (*e.g.* aqueous solution or within a protein)^40^ and the dynamics of the photoactive compound.

### Substituent-induced exciton localization (SIEL) on other Ru systems

**Fig. 7:**
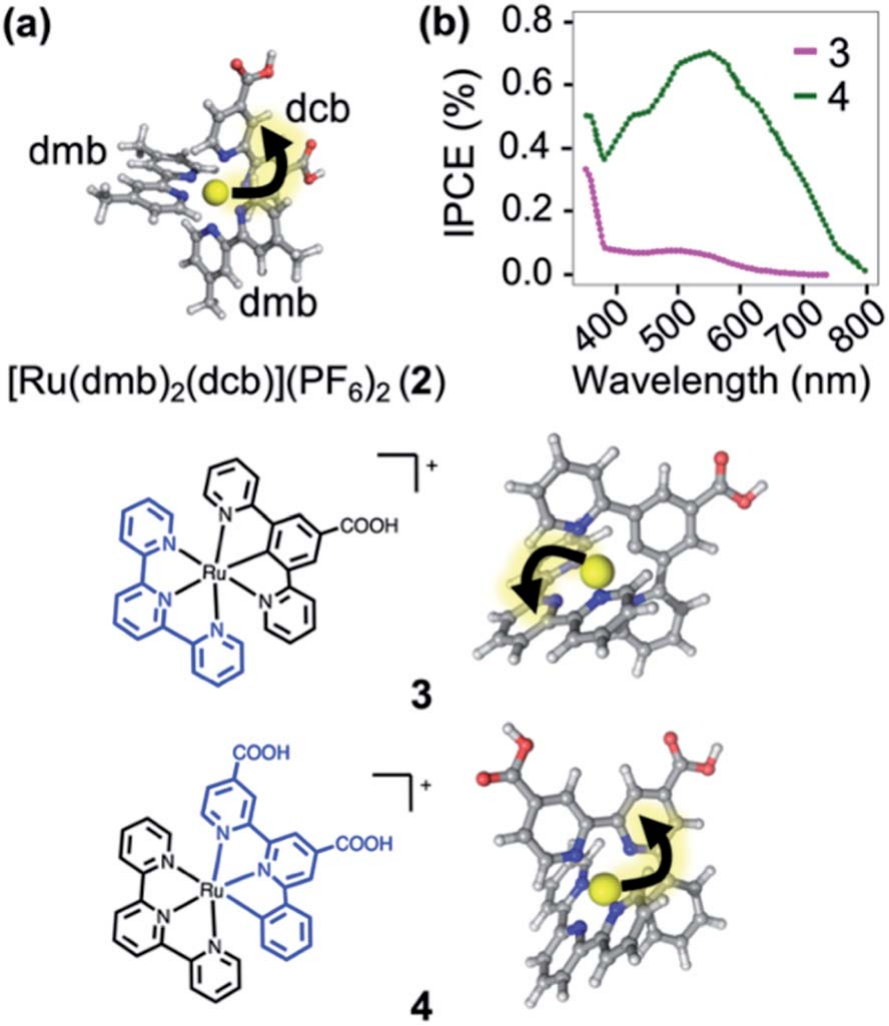
(a) [Ru(dmb)_2_(dcb)]^2+^ (**2**), where dmb = 4,4′-dimethyl-2,2′-bipyridine and dcb = 4,4′-dicarboxylic-2,2′-bipyridine. (b) Plot of the prediction of “incident photon to current efficiency” (IPCE) into TiO_2_, adapted from ref. 42. Below are shown the carbometallated Ru-complexes^42^**3** and **4** decorated with a 2,2′:6′,2′′-terpyridine (tpy) ligands (left) and the directionality of the excited electron highlighted with an arrow (right).

In order to illustrate the predictive power of the SIEL descriptor, we apply this concept to several systems previously characterized experimentally. The first is [Ru(dmb)_2_(dcb)]^2+^ (**2**), which has been extensively used as molecular dye to photosensitize TiO_2_ surfaces.^41^**2** is decorated with two methyl groups on the dimethylbipyridine dmb units (four in total) and with two carboxylic acid groups in the dicarboxylicbipyridine dcb unit (Fig. 7a). The latter are used to bind to the metallic surface and to injects electrons, after excitation and subsequent population of the lowest excited ^3^MLCT state. By quickly adding up the tabulated SIEL values for the introduction of a methyl group (0.26) and a carboxylic acid group (–0.71) in the ^3^MLCT state (Table S5†), the estimated net SIEL value on each of the two dcb ligands is –1.42 (attraction of the excited electron) and 0.52 in dmb (repulsion). It can therefore be concluded that the excitation will clearly happen to dcb, and thus in the direction of the TiO_2_ surface. An explicit calculation of the SIEL values more accurately in **2** using *ab initio* computations leads to the same conclusion (Tables S6 and S7†). Importantly, this tell us that the computed values displayed in Fig. 4 can be used to quickly anticipate the effect of single modifications on C-4 atoms of bpy units of [Ru(bpy)_3_]^2+^.

The applicability of SIEL to other chemical configurations is illustrated next by rationalizing the origin of the discrepancies in the photocurrent action spectra/sensitizing properties between the two cyclometalated complexes [Ru((COOH)-N∧C∧N)(tpy)]^+^ (**3**) and [Ru((COOH)_2_-C∧N∧N)(tpy)]^+^ (**4**)^42^ (Fig. 7b). Both pigments for dye-sensitized solar cells have been also anchored on a TiO_2_ surface through their carboxylic acid groups^55^,^56^ to generate currents upon light absorption by injection of the excited electron into the metal support. However, complexes **3** and **4** differ in their scaffolds. Van Koten and colleagues^42^ found that the nature of the excited state highly depends on the complex, affecting their proficiency as photosensitizer. Fig. 7b shows that the experimental photocurrent action spectra^42^ of **3** (magenta line) is less intense than that of **4** (green line). We computed the SIEL values on their lowest excited triplet states using three fragments (Table S9†). Based on our analysis, in the lowest ^3^MLCT excited state of the less active compound **3**, the excited electron is transferred to the distal 2,2′:6′,2′′-terpyridine (tpy) unit (SIEL = –0.415) with respect to the TiO_2_ surface. On the contrary, in the more active compound **4**, the excited electron localizes on the ligand that carries the carboxylic acids ((COOH)_2_-C∧N∧N, SIEL = –0.376) and, therefore, electron transfer from that ligand to the surface is highly favored. Both cases feature a ^3^MLCT excited triplet, but they possess a non-negligible ligand-to-ligand charge transfer (LLCT) contribution, which is almost double in **3** than in **4** (Table S8†).

**Scheme 2:**
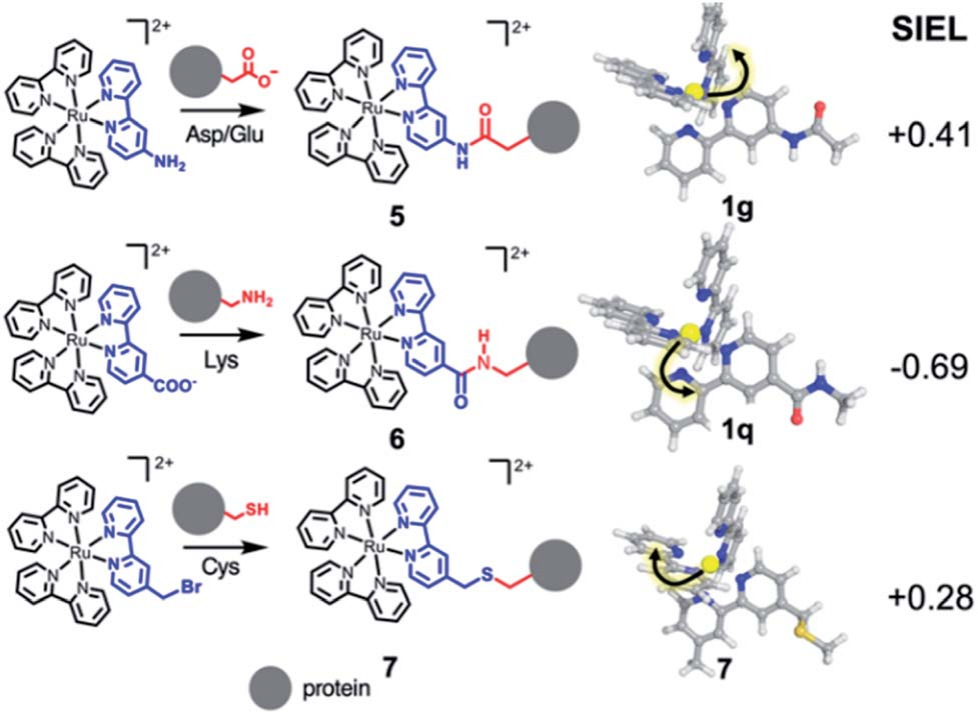
Excited electron directionality in covalent-bound Ru-based complexes within protein environments. Ru-based photosensitizers **5–7** bound to an amino acid, showing directional excitation on the lowest ^3^MLCT state (geometries shown in sticks) based on the SIEL values.

As third example, we analyzed three Ru–diimine complexes covalently bound to proteins – one of the most explored avenues to couple light and enzyme activities by mediation of electron transfer processes.^43^,^57^ As main strategy, a synthetic Ru-based polypyridine species is reacted with the side chain of an amino acid that is positioned in proximity to the active site or to the place where the electron transfer process happens (Scheme 2). Frequently targeted residues are Asp, Glu or Lys, *via* an amide bond (compounds **5** and **6**, respectively), or Cys,^43^ connected through a thioether group (compound **7**). The different nature of the residue is expected to affect the localization of the excited electron. Since the kinetics of the electron transfer process depends, among others, on the distance between donor and acceptor,^58^ the electron transfer processes could be modulated by the quantification of the SIEL values on the photosensitizer and the proper orientation of the coordination complex with respect to the acceptor species.

According to the SIEL values for relaxed ^3^MLCT states tabulated in Fig. 4, NHCOCH_3_ (**1g**) would repel the excited electron and would trigger the excitation into the ligand in *cis* (**5**, SIEL = 0.41). In contrast, the use of CONHCH_3_ (**1q**) would attract the excited electron to the ligand where the functional group is located (**6**, SIEL = –0.69). Coupling with a Cys residue through a thioether group (**7**) will play the same role, repelling the excited electron from the ligand that binds to the protein (SIEL = 0.28) although to the *trans* bpy unit (Tables S10 and S11†). We see thus that the selection of a particular chemical linker changes the directionality of the excited electron and the use of SIEL can predict it, to the advantage of the experimental setup.^44^

**Scheme 3:**
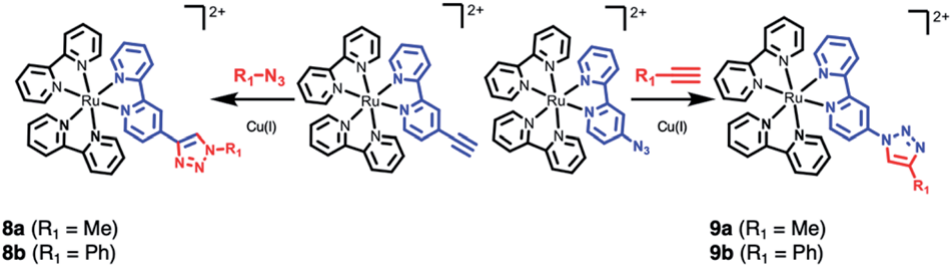
CuAAC (Cu(i) catalyzed azide–alkyne cycloaddition) click chemistry adducts **8** and **9**.^45^,^46^

The final example consists of two click-chemistry products, **8** and **9**, synthesized from the precursors [Ru(bpy)_2_(bpy-CCH)]^2+^ and [Ru(bpy)_2_(bpy-N_3_)]^2+^, respectively (Scheme 3), and described by Aukauloo *et al.*^45^,^46^ Since the number of proteinogenic residues is limited, different approaches emerged in the last years to expand the chemical reactivity space of the amino acids. One of them is the well-known click chemistry methodology,^59^,^60^ that allows a modular approach within Chemical Biology. In particular, the CuAAC (Cu(i) catalyzed azide–alkyne cycloaddition) click chemistry only requires the presence of two reactants, an alkyne and an azide species, together with Cu(i) as catalyst. The chemical stability of these species allows that the click reaction can be carried out *in vivo* in a mild manner. This triazole group can be a useful linker for electron-transfer processes. Some examples have been developed using [Ru(bpy)_3_]^2+^ as scaffold.^45^,^46^,^61^,^62^ In particular, we have studied the effect of a methyl group (R_1_ = Me, **8a** and **9a**) and a phenyl group (R_1_ = Ph, **8b** and **9b**) on the triazole moiety (Scheme 3, Tables S12 and S13†). In contrast to the experimental observation that the 1,2,3-triazoles in **8** and **9** are electrochemically silent and do not alter the intrinsic photophysical properties of the Ru-based chromophore in solution,^45^,^46^ the nature of the triazole group is expected to affect the exciton directionality.

The SIEL numbers for bpy1-R of **8** and **9** are shown in Table 1. We see that whereas in the two derivatives of **9** the 1,2,3-triazole attracts the excited electron to bpy1 (SIEL = –0.656 (Me) and –0.497 (Ph)), the former heterocycle repels the excitations in **8a**/**b** to bpy2/bpy3 (SIEL = 0.345 (Me) and 0.039 (Ph)). Thus, the nature of triazole change the directionality of the excitation (**8a***vs.***9a**, **8b***vs.***9b**) but the change Me → Ph in **8** and/or in **9** affects in less extend the directionality of the excitation (**8a***vs.***8b**, **9a***vs.***9b**).

**Table 1:** SIEL of the excited electron in the triazole-substitute fragment (bpy1) and excited electron population per fragment in the lowest excited ^3^MLCT state of complexes **8** and **9**

Cmp.	R_1_	SIEL	Excited electron population^*a*^ (%)			
			1,2,3-triazole-R_1_	bpy_1_^*a*^	bpy_2_ (*trans*)	bpy_3_ (*cis*)
**8a**	Me	0.345	4	4	79	13
**9a**	Me	–0.656	3	75	11	11
**8b**	Ph	0.039	13	17	16	54
**9b**	Ph	–0.497	15	54	14	17

^*a*^For the calculation of the excited electron populations, the complexes have been divided into 5 fragments (Ru, bpy1, triazole, bpy2 and bpy3). *trans* and *cis* refer to the relative position of the bpy unit to the substituted one. The excited electron population on Ru is 0% in all cases.

In addition to the calculations done with 4 fragments, we also split the system into 5 fragments to compute separately the excited electron population in the bare bpy1 and in the substituted triazole with the functional group R_1_ (Table 1). As an example, we can see that, whereas in **8a** (R = Me) 8% of the population of the excited electron is located on bpy1 + R_1_, the introduction of a phenyl ring in **8b** (R = Ph) increases this population only up to a 30%. In the change **9a** → **9b** the effect on the Ph ring has a smaller impact on the population on bpy1 + R_1_ of the N-connected triazole (78% *vs.* 69%).

## Conclusions

We demonstrate that the substituent-induced exciton localization (SIEL) descriptor, combined with the excited-electron delocalization length (EEDL_*n*_) is a powerful tool to quantify exciton directionality and localization, paving the way for easy rational design of photosensitizers. SIEL, as well as EEDL_*n*_, are implemented to be universally used on any chemical system in a black-box fashion, rendering a straightforward quantification of the effect of chemical modifications on electronic excitations. No visual inspection of molecular orbitals is necessary, eliminating sources of bias and subjectivity. The achieved quantification proves particularly advantageous when large ensembles of molecules are considered, for instance to take into account environment, or in data mining studies, where increasingly larger sets of generated data can reveal important features if appropriate quantitative measures are available.

As proof of concept, we quantified the effect of 22 chemical modifications on the archetype [Ru(bpy)_3_]^2+^ and rationalized the directionality of the excitation in four experimental cases with technological and biological relevance: photosensitizers for solar cells, metalloproteins for enzymatic photocatalysis and click chemistry for biological labeling – processes where the directional electron transfer is key for the overall photoinduced mechanism. We proved that our descriptors can, (i) help in the design of directional electron transfer within chromophores attending to their chemical functionalization, and (ii) explain experimental trends. We thus expect these descriptors to become a valuable tool to design new photosensitizers with improved electronic properties.

## Data availability

The research data supporting this publication can be accessed *via* the institutional open repository of the University of Vienna ; https://doi.org/11353/10.1032866. The code for the 1TDM analysis can be found at ; theodore-qc.sourceforge.net.

## Conflicts of interest

There are no conflicts to declare.

## Supplementary Material

Supplementary information
