# Supplementary material for: Orbital-free photophysical descriptors to predict directional excitations in metal-based photosensitizers
Source: Chem Sci. 2020 May 15;11(29):7685–93. doi: 10.1039/d0sc01684e (PMC7425079; doi:10.1039/d0sc01684e)
Supplement: Supplementary file 1 [file SC-011-D0SC01684E-s001.pdf]

Electronic Supplementary Material (ESI) for Chemical Science.
This journal is © The Royal Society of Chemistry 2020

Supporting Information

Orbital-free photophysical descriptors to predict directional excitations in metal-based photosensitizers

Pedro A. Sánchez-Murcia,[a]* Juan J. Nogueira,[b] Felix Plasser[c] and Leticia González[a,d]*

[a]*Institute of Theoretical Chemistry, Faculty of Chemistry, University of Vienna, Währinger Str. 17, 1090 Vienna, Austria, pedro.murcia@univie.ac.at, leticia.gonzalez@univie.ac.at*

[b]*Departamento de Química and Institute for Advanced Research in Chemistry, Universidad Autónoma de Madrid, 28049, Madrid, Spain*

[c]*Department of Chemistry, Loughborough University, LE11 3TU, Loughborough, United Kingdom.*

[d]*Vienna Research Platform for Accelerating Photoreaction Discovery, University of Vienna, Währinger Str. 17, 1090 Vienna, Austria*

Table S1 CAS numbers and references of the complexes **1a-1v** studied in this work S3

Table S2 Averaged $EEDL_n$ (%) values of the initially excited singlet ^{1}MLCT state in **1a-1v** S4

Table S3 Averaged $EEDL_n$ (%) values of the relaxed lowest excited triplet ^{3}MLCT state in **1a-1v** S5

Table S4 Energy of the three local minima (in Hartrees, H) for the excited triplet state and Boltzmann partition factors q_i ($e\Delta Ei/kBT / \sum e\Delta Ei/kBT$) of compounds **1a**-**1v** S6

Table S5 Averaged SIEL values of both initial (^{1}MLCT) and relaxed (^{3}MLCT) states of compounds **1a**-**1v**, Hammet (σ_p), modified Swain-Lupson (F, R, R^+) and Brown-Okamoto (σ_p^+) parameters S7

Tables S6 and S7 Example 1: $[Ru(dmb)_2(dcb)]^{2+}$ (**2**) S8

Tables S8 and S9 Example 2: Carbometallated Ru-complexes **3** and **4** S9

Tables S10 and S11 Example 3: Thioeter **7** S10

Tables S12 and S13 Example 4: Click-chemistry products **8** and **9** S11

Section S1 Computational methods S12

Section S2 Computational protocol to compute $EEDL_n$ and SIEL descriptors S13

References S15

**Table S1** CAS numbers and references of the complexes **1a-1v** studied in this work.			
**Compound**	**R**	**CAS Nr.**	**Reference/Comments**
**1a**	H (prototype)	15158-62-0	
**1b**	F	(1538613-86-3, heteroleptic complex)	No substitution on bpy, but with 2F atoms on TAP
**1c**	Cl	202644-88-0	
**1t**	CH_3	218601-05-9	*Australian J. Chem.* **1998**, *51*, 999-1002
**1h**	$N(CH_3)_3^+$	- (meta: -, ortho: -)	
**1s**	CN	835604-82-5	Inorg. Chem. **2005**, *44*, 5-7
**1d**	CF_3	2135655-10-4	*J. Am. Chem. Soc.* **2017**, *139*, 15347
**1u**	Ph	1073441-12-9	*J. Electrianal. Chem.* **2008**, *621*, 277
**1i**	NO_2	75777-81-0	*Isr. J. Chem.* **1982**, *22*, 89-90
**1e**	NH_2	752164-70-8, 878047-53-1	*Inorg. Chem.* **2012**, *51*, 5985, *Polyhedron* **2006**, *25*, 437
**1k**	OCH_3	701902-98-9	*Monatshefte für Chemie* **1988**, *119*, 1-15
**1j**	OH	878047-54-2	*Polyhedron* **2006**, *25*, 437
**1f**	$NHCH_3$		No monoalkyl, but longer alkyl groups (dimethyl)
**1g**	$NHCOCH_3$	(with TAP) 478554-95-9 (diAcN)	*J. Am. Chem. Soc.* **2002**, *124*, 13694
**1l**	CHO	(with methyl 150430-57-2, or dialdehyde)	*Inorg. Chem.* **2017**, *56*, 3794 *Chemistry* **2016**, *22*, 9649
**1m**	$C(O)CH_3$	(acac 473546-20-2 or longer 72077-46-4)	*J. Am. Chem. Soc.* **2002**, *124*, 11541
**1n**	COOH	218600-82-9	EPC 2016 3108900 *Inorg. Chem.* **2005**, *44*, 8317, *Australian J. Chem.* **1988**, *51*, 999
**1o**	$COOCH_3$	1219930-83-2	*Organometallics* **2010**, *29*, 1569
**1r**	$COCHCH_2$	-	
**1v**	SO_2CH_3	165901-10-0	*Inorg. Chem.* **1995**, *34*, 2759

Table S2 Averaged $EEDL_n$ (%) values of the initially excited singlet ^1MLCT state in **1a-1v**.

**Compound**	EELD (%)			
	**$EEDL_1$**	**$EEDL_2$**	**$EEDL_3$**	**$EEDL_4$**
**1a** (H)	12	40	41	5
**1b** (F)	15	39	41	5
**1c** (Cl)	15	39	41	5
**1d** (CF_3)	23	39	34	4
**1e** (NH_2)	18	46	34	2
**1f** ($NHCH_3$)	19	45	35	1
**1g** ($NHCOCH_3$)	17	44	38	1
**1h** ($N(CH_3)_3^+$)	34	36	26	4
**1i** (NO_2)	29	40	30	1
**1j** (OH)	17	40	41	2
**1k** (OCH_3)	15	46	37	2
**1l** (CHO)	22	41	34	3
**1m** ($C(O)CH_3$)	19	42	36	4
**1n** (COOH)	15	41	41	4
**1o** ($COOCH_3$)	21	42	34	3
**1p** ($CONH_2$)	20	44	35	1
**1q** ($CONHCH_3$)	22	45	32	1
**1r** ($COCHCH_2$)	25	46	29	1
**1s** (CN)	14	79	7	0
**1t** (CH_3)	15	43	40	2
**1u** (Ph)	17	45	37	1
**1v** (SO_2CH_3)	22	47	30	1

**Table S3** Averaged $EEDL_n$ (%) values of the relaxed lowest excited triplet ^{3}MLCT state in **1a-1v**.				
**Compound**	EELD (%)			
	**$EEDL_1$**	**$EEDL_2$**	**$EEDL_3$**	**$EEDL_4$**
**1a** (H)	77	20	0	3
**1b** (F)	89	9	0	2
**1c** (Cl)	89	9	0	2
**1d** (CF_3)	89	9	0	2
**1e** (NH_2)	80	17	0	3
**1f** ($NHCH_3$)	81	16	0	3
**1g** ($NHCOCH_3$)	90	7	0	3
**1h** ($N(CH_3)_3^+$)	97	0	0	3
**1i** (NO_2)	94	3	0	3
**1j** (OH)	86	11	0	3
**1k** (OCH_3)	77	20	0	3
**1l** (CHO)	79	18	0	3
**1m** ($C(O)CH_3$)	81	14	0	5
**1n** (COOH)	80	17	0	3
**1o** ($COOCH_3$)	81	19	0	0
**1p** ($CONH_2$)	65	32	0	3
**1q** ($CONHCH_3$)	79	17	0	4
**1r** ($COCHCH_2$)	68	29	0	3
**1s** (CN)	94	3	0	3
**1t** (CH_3)	86	11	0	3
**1u** (Ph)	79	15	3	3
**1v** (SO_2CH_3)	89	9	0	2

**Table S4** Energy of the three local minima (in Hartrees, H) for the excited triplet state and Boltzmann partition factors q_i ($e\Delta Ei/kBT / \sum e\Delta Ei/kBT$) of compounds **1a**-**1v**.						
**Compound**	E_1 (H)	E_2 (H)	E_3 (H)	q_1	q_2	q_3
**1a** (H)	-1580.751127	-	-	0.33	0.33	0.33
**1b** (F)	-1680.036738	-1680.035557	-1680.035269	0.67	0.19	0.14
**1c** (Cl)	-2040.382750	-2040.381566	-2040.381286	0.67	0.19	0.14
**1d** (CF_3)	-1917.932033	-1917.927033	-1917.927033	0.99	0.01	0.01
**1e** (NH_2)	-1636.136689	-1636.132709	-1636.136675	0.50	0.01	0.49
**1f** ($NHCH_3$)	-1675.432539	-1675.427189	-1675.432374	0.54	0.00	0.46
**1g** ($NHCOCH_3$)	-1788.810524	-1788.807386	-1788.813724	0.03	0.00	0.97
**1h** ($N(CH_3)_3^+$)	-1754.205208	-1754.203152	-1754.203152	0.81	0.09	0.09
**1i** (NO_2)	-1785.326743	-1785.326760	-1785.326759	0.33	0.34	0.34
**1j** (OH)	-1656.010167	-1656.006891	-1656.008707	0.80	0.03	0.17
**1k** (OCH_3)	-1695.300089	-1695.295695	-1695.300090	0.50	0.00	0.50
**1l** (CHO)	-1694.107034	-1694.100043	-1694.101220	1.00	0.00	0.00
**1m** ($C(O)CH_3$)	-1733.418922	-1733.414051	-1733.416168	0.94	0.01	0.05
**1n** (COOH)	-1769.391136	-1769.387389	-1769.386654	0.97	0.02	0.01
**1o** ($COOCH_3$)	-1808.681020	-1808.677994	-1808.678272	0.91	0.04	0.05
**1p** ($CONH_2$)	-1749.499504	-1749.495264	-1749.498241	0.78	0.01	0.21
**1q** ($CONHCH_3$)	-1788.797451	-1788.793521	-1788.79352	0.97	0.02	0.02
**1r** ($COCHCH_2$)	-1771.504657	-1771.505599	-1771.507538	0.04	0.11	0.85
**1s** (CN)	-1673.017561	-1673.010047	-1673.009883	1.00	0.00	0.00
**1t** (CH_3)	-1618.199976	-1618.200056	-1618.198194	0.45	0.49	0.07
**1u** (Ph)	-1811.818961	-1811.817272	-1811.818950	0.46	0.08	0.46
**1v** (SO_2CH_3)	-2168.745388	-2168.740766	-2168.740767	0.98	0.01	0.01

**Table S5** Averaged SIEL values of both initial (^1MLCT) and relaxed (^3MLCT) states of compounds **1a**-**1v**, Hammet (σ_p), modified Swain-Lupson (F, R, R^+) and Brown-Okamoto (σ_p^+) parameters obtained from the reference.[1]								
**Compound / R**		σ_p	σ_p^+	**SIEL(1)**	**SIEL(2)**	**F**	**R**	R^+
**1a**	H	0.0	0.0	0.02	0.00	0	0	0
**1b**	F	0.06	-0.07	-0.02	-0.40	0.45	-0.39	-0.52
**1c**	Cl	0.23	0.11	-0.05	-0.46	0.42	-0.19	-0.31
**1d**	CF_3	0.54	0.61	-0.14	-0.81	0.38	0.16	0.23
**1e**	NH_2	-0.66	-1.30	0.09	0.39	0.08	-0.74	-1.38
**1f**	NHR	-0.70	-1.81	0.08	0.41	-0.03	-0.73	-1.73
**1g**	$NHCOCH_3$	0.00	-0.60	0.03	0.41	0.31	-0.31	-0.91
**1h**	$N(CH_3)^+$	0.60	0.41	-0.24	-0.86	0.92	-0.32	-0.45
**1i**	NO_2	0.78	0.79	-0.17	-0.86	0.65	0.13	0.14
**1j**	OH	-0.37	-0.92	0.03	0.38	0.33	-0.70	-1.25
**1k**	OCH_3	-0.27	-0.78	0.06	0.40	0.29	-0.56	-1.07
**1l**	CHO	0.42	0.73	-0.13	-0.79	0.33	0.09	0.40
**1m**	$C(CH_3)O$	0.50	-(3)	-0.07	-0.68	0.33	0.17	0.17
**1n**	COOH	0.45	0.42	-0.06	-0.71	0.34	0.11	0.08
**1o**	COOR	0.45	0.49	-0.12	-0.64	0.34	0.11	0.14
**1p**	$CONH_2$	0.36	-(3)	-0.03	-0.48	0.26	0.10	0.10
**1q**	$CONHCH_3$	0.36	-(3)	-0.05	-0.66	0.35	-0.01	-0.01
**1s**	CN	0.66	0.66	-0.20	-0.80	0.51	0.15	0.15
**1t**	CH_3	-0.17	-0.31	-0.04	-0.83	0.01	-0.18	-0.32
**1u**	Ph	-0.01	-0.18	0.01	0.26	0.12	-0.13	-0.30
**1v**	SO_2CH_3	0.72	-(3)	-0.10	0.36	0.59	0.18	0.18
(1)Averaged value computed over 2500 states with more than 50% MLCT character. (2)Averaged value computed over ca. 100 excited states with more than 50% MLCT character. (3)Data not available.								

Example 1: [Ru(dmb)$_2$(dcb)]$^{2+}$ (2)[2]

Table S6. CT character (%) and individual components for the lowest triplet excited state of **2**.[(1)]					
**CT**	**MLCT**	**LLCT**	**LMCT**	**LC**	**MC**
87	71	15	2	7	5
[(1)]Computed on 100 excited states.					

Table S7. SIEL values for bpy1, bpy2 and bpy3 in the lowest triplet excited state of **2**.[(1)]		
**bpy1-FG**	**bpy2**	**bpy3**
-0.78	0.36	0.42
[(1)]Computed with 100 excited states.		

Example 2: carbometallated Ru-complexes 3 and 4[3]

Table S8. CT character (%) and individual components for the lowest triplet excited state of **3** and **4**.[(1)]

Compound	CT	MLCT	LLCT	LMCT	LC	MC
**3**	86	43	37	5	8	5
**4**	65	40	20	5	30	5

[(1)]Computed with 100 excited states.

Table S9. SIEL values for ligand 1 and ligand 2 in the lowest triplet excited state of **3** and **4**.[(1)]

Compound	ligand1	ligand2 (w/ COOH)
1	-0.42	+0.42
2	+0.38	-0.38

[(1)]Computed with 100 excited states.

Example 3: thioeter 7

Table S10. CT character (%) and individual components for the lowest triplet excited state of **7**.[1]					
**CT**	**MLCT**	**LLCT**	**LMCT**	**LC**	**MC**
24	18	3	4	69	6

[1]Computed on 100 excited states.

Table S11. SIEL values for bpy1, bpy2 and bpy3 in the lowest triplet excited state of **7**.[1]		
**bpy1-FG**	**bpy2**	**bpy3**
0.28	0.12	-0.34

[1]Computed with 100 excited states.

Example 4: Click-chemistry products 8 and 9[4,5]

**Table S12.** CT character (%) and individual components for the lowest triplet excited state of **8** and **9**.[(1)]						
**Compound**	**CT**	**MLCT**	**LLCT**	**LMCT**	**LC**	**MC**
**8a**	85	60	25	2	12	3
**8b**	19	15	2	2	76	5
**9a**	68	50	16	2	3	2
**9b**	17	12	3	21	81	3
[(1)]Computed with 100 excited states.						

**Table S13.** SIEL values for bpy1, bpy2 and bpy3 in the lowest triplet excited state of **8** and **9**.[(1)]			
**Compound**	**bpy-triazole**	**bpy2**	**bpy3**
**8a**	0.345	-0.628	0.283
**8b**	0.039	0.247	-0.286
**9a**	-0.656	0.328	0.328
**9b**	-0.497	0.275	0.222
[(1)]Computed with 100 excited states.			

Section S1 Computational methods

Geometry optimization of the ground and triplet excited states. Cartesian coordinates of **1a** were obtained from the Cambridge Structural Database (CSD) (id. BPYRUF).[6] Initial guesses for the structure of complexes **1b-1v** were built from the prototype **1a** by replacement of a hydrogen atom with the corresponding functional group. Ground-state geometry optimization of complexes **1a-1v** was done in gas phase using density functional theory (DFT) with the hybrid functional B3LYP.[7] The energy was corrected by the original D3 damping function of Grimme.[8] The def2-svp basis set[9,10] was used on the C, H, N, S and O atoms and Stuttgart relativistic small-core effective potential (MWB28) was used to described the 28 core electrons of the Ru atom.[11] No symmetry criterion was imposed. Triplet-excited state geometries were optimized with the same hybrid functional but with a bigger basis set for the nonmetallic atoms (def2-TZVP). All the calculations were carried out with Gaussian 16, revision A.03.[12]

Conformational and vibrational sampling. Harmonic vibrational frequencies were computed from the corresponding optimized geometry at the same level of theory as geometry optimization. These frequencies were used to compute a quantum-harmonic Wigner distribution with finite-temperature correction.[13,14] 100 geometries were computed for each of the optimized geometries of **1a-1v**. The Boltzmann-weighted distribution was computed at 300 K and the energies and derivatives of energies were not scaled.

Linear response TD-DFT/TDA. For the 100 geometries around the optimized ground state geometry of each of the derivatives, the first 25 singlet states of the metal complex were computed by linear response TD-DFT/TDA (2500 total excited states per derivative). For these excited singlet states, the hybrid functional B3LYP was selected. For the first triplet excited state, the standard XC PBE functional was used, since it has been shown that this functional reproduces the state character and ordering in closer agreement to MS-CASPT2 than the hybrid functionals on Ru(II) complexes.[15] In the case of the lowest triplet excited state, we explored two additional local minima by substituting the other two units on the optimized geometry of the lowest triplet state. The computation of the Boltzmann factors showed that in all cases the three local minima were close in energy. Therefore, we computed the SIEL value of the excited triplet state as a weighted average value of the three states, with a total of 100 excited states per derivative (see Table S5). For all the excited states (singlets and triplets), the same basis set (def2-TZVP) was used. Additionally, in those systems, where excitations over 5 Å were observed (compounds **3**, **4**, **7**, **8** and **9**), the long-range correction scheme of Hirao *et al.*[16] was applied in combination with the former GGA functional (LC-PBE). Although not applied here, the effect of the environment could be included in $EEDL_n$ and SIEL by means of representing it as point charges in these static TD-DFT calculations.

Section S2 Computational protocol to compute $EEDL_n$ and SIEL numbers

The calculation of both descriptors has been implemented in TheoDORE[17] and can be easily executed in an automatic fashion in any computer with UNIX system. An additional QM software capable of running excited states calculations is required. In general, the following steps are required:

1. *Quantum mechanics (QM) calculation 1: Geometry optimization*.[(*)] The cartesian coordinates of the system of study can be obtained from experimental databases like The Cambridge Structural Database (CSD, https://www.ccdc.cam.ac.uk). If not, an initial guess can be generated using any computational tool or graphical interface (e.g. Avogadro, ChemBioDraw, Molden, Corina3D, …). After that, the system can be optimized using any quantum chemical method, supported by your favorite QM software. In this work we optimized the geometry of the metal complexes at the B3LYP-D3/MWB28(Ru)/def2-svp level of theory using the software package Gaussian 16, revision A.03.[12]
2. *Quantum mechanics (QM) calculation 2: Excited states calculation*. After the geometry optimization, the excited states calculation (e.g. using TD-DFT) can be run with any standard QM software compatible with TheoDORE,[17] see https://sourceforge.net/p/theodore-qc/wiki/Program%20specific%20information/. The number and multiplicity of the excited states will be specified in this step. IMPORTANT: in order to compute the transition density matrix by TheoDORE in the next step, specific flags have to be included in the input file of the QM program. Check in the *Program specific information* part of the former link. The output file of the QM program will be required for the next step.

3. *Fragments definition and wave function analysis with TheoDORE.*[17] The input file for TheoDORE can be easily generated with the *theoinp* module (https://sourceforge.net/p/theodore-qc/wiki/Input/). In this step the fragment definition of the system is setup (flag *at_lists*). To compute the two novel parameters, SIEL and PR_f have to be specified in the field *prop_lists* of the input file. Next, the module *analyze_tden.py* will run the 1TDM calculation and the parameters derived from that. The value of SIEL will be obtained for each of the excited states as well as the PR_f value. For computing the $EEDL_n$ of an ensemble of excited states, Eq. 2 should be followed. As example, the use of the *spectrum.py* could be used for this purpose.

This process can be fully automatized for research campaigns as that shown in this manuscript for the $[Ru(bpy)_3]^{2+}$ derivatives. (*) NOTE: In order to get an ensemble of conformers for a particular compound -as we did in this manuscript with all the examples – an additional step would be required before step 2 for the conformational/vibrational phase space sampling. We encourage the reader to include this step to get significant results for $EEDL_n$. One approach consists of a frequencies calculation using a QM program and, with the output

of this calculation, the input geometries for step 3 (e.g. 100 conformers) by means of a Wigner distribution (e.g. we used the freely-available module *wigner.py* implemented in SHARC).[18] Another option would be the thermal sampling of the system of study by means of a QM/MM MD simulation as described elsewhere.[19] The latter possibly would allow the inclusion of environmental effects into the calculations.

References

1 C. Hansch, A. Leo and R. W. Taft, *Chem. Rev.*, 1991, **91**, 165–195.
2 H. Y. Chen and S. Ardo, *Nat. Chem.*, 2018, **10**, 17–23.
3 S. H. Wadman, J. M. Kroon, K. Bakker, R. W. A. Havenith, G. P. M. Van Klink and G. Van Koten, *Organometallics*, 2010, **29**, 1569–1579.
4 A. Baron, C. Herrero, A. Quaranta, M. F. Charlot, W. Leibl, B. Vauzeilles and A. Aukauloo, *Chem. Commun.*, 2011, **47**, 11011–11013.
5 A. Baron, C. Herrero, A. Quaranta, M. F. Charlot, W. Leibl, B. Vauzeilles and A. Aukauloo, *Inorg. Chem.*, 2012, **51**, 5985–5987.
6 P. Rillema and D. S. Jones, *Chem. Commun.*, 1979, 849–851.
7 A. D. Becke, *J. Chem. Phys.*, 1993, **98**, 1372–1377.
8 S. Grimme, J. Antony, S. Ehrlich and H. Krieg, *J. Chem. Phys.*, 2010, **132**, 154104.
9 F. Weigend and R. Ahlrichs, *Phys. Chem. Chem. Phys.*, 2005, **7**, 3297–3305.
10 F. Weigend, *Phys. Chem. Chem. Phys.*, 2006, **8**, 1057–1065.
11 D. Andrae, U. Häußermann, M. Dolg, H. Stoll and H. Preuß, *Theor. Chim. Acta*, 1990, **77**, 123–141.
12 Gaussian 16, Revision A.03, M. J. Frisch, G. W. Trucks, H. B. Schlegel, G. E. Scuseria, M. A. Robb, J. R. Cheeseman, G. Scalmani, V. Barone, G. A. Petersson, H. Nakatsuji, X. Li, M. Caricato, A. V. Marenich, J. Bloino, B. G. Janesko, R. Gomperts, B. Mennucci, H. P. Hratchian, J. V. Ortiz, A. F. Izmaylov, J. L. Sonnenberg, D. Williams-Young, F. Ding, F. Lipparini, F. Egidi, J. Goings, B. Peng, A. Petrone, T. Henderson, D. Ranasinghe, V. G. Zakrzewski, J. Gao, N. Rega, G. Zheng, W. Liang, M. Hada, M. Ehara, K. Toyota, R. Fukuda, J. Hasegawa, M. Ishida, T. Nakajima, Y. Honda, O. Kitao, H. Nakai, T. Vreven, K. Throssell, J. Montgomery, J. A., J. E. Peralta, F. Ogliaro, M. J. Bearpark, J. J. Heyd, E. N. Brothers, K. N. Kudin, V. N. Staroverov, T. A. Keith, R. Kobayashi, J. Normand, K. Raghavachari, A. P. Rendell, J. C. Burant, S. S. Iyengar, J. Tomasi, M. Cossi, J. M. Millam, M. Klene, C. Adamo, R. Cammi, J. W. Ochterski, R. L. Martin, K. Morokuma, O. Farkas, J. B. Foresman and D. J. Fox, Gaussian, Inc., Wallingford CT, 2016.
13 J. P. Dahl and M. Springborg, *J. Chem. Phys.*, 1988, **88**, 4535–4547.
14 J. P. Zobel, J. J. Nogueira and L. González, *Phys. Chem. Chem. Phys.*, 2019, **21**, 13906–13915.
15 A. J. Atkins, F. Talotta, L. Freitag, M. Boggio-Pasqua and L. González, *J. Chem. Theory Comput.*, 2017, **13**, 4123–4145.
16 H. Iikura, T. Tsuneda, T. Yanai and K. Hirao, *J. Chem. Phys.*, 2001, **115**, 3540–3544.
17 F. Plasser, *J. Chem. Phys.*, 2020, **152**, 1–14.
18 SHARC 2.0: Surface Hopping Including Arbitrary Couplings – Program Package for Non-Adiabatic Dynamics, S. Mai, M. Richter, M. Heindl, M. Menger, A. Atkins and M. Ruchenbauer, University of Vienna, Vienna, 2019.
19 P. A. Sánchez-Murcia, J. J. Nogueira and L. González, *J. Phys. Chem. Lett.*, 2018, **9**, 683–688.
